# One-year follow-up of changes in refraction and aberrations induced by corneal incision

**DOI:** 10.1371/journal.pone.0224823

**Published:** 2019-11-18

**Authors:** Eloy A. Villegas, Encarna Alcón, Elena Rubio, José María Marín, Pablo Artal

**Affiliations:** 1 Laboratorio de Óptica, Universidad de Murcia, Murcia, Spain; 2 Servicio de Oftalmología, Hospital Virgen de la Arrixaca, Murcia, Spain; Nicolaus Copernicus University, POLAND

## Abstract

**Purpose:**

To evaluate the surgically induced changes in refraction (sphere and astigmatism) and higher order aberrations by corneal incision for one year.

**Setting:**

University Hospital “Virgen de la Arrixaca”, Murcia, Spain.

**Design:**

Retrospective interventional case series.

**Methods:**

Corneal power, astigmatism and higher order aberrations (HOA) were calculated from corneal topography measured in 27 eyes prior to surgery and at 2 weeks, 1, 2, 3 and 6 months and 1 year following cataract surgery with 3.2-mm corneal incision. At every stage, optical changes were calculated as the difference between pre- and post-surgery data (in each follow-up) using the formulas of obliquely crossed cylinders for the refraction and Zernikes coefficients for HOA.

**Results:**

At 2 weeks after surgery the mean corneal values of induced sphere, cylinder and the root mean square (RMS) of HOA were +0.54±0.27 D, -0.77±0.32 D and 0.15 microns respectively. These parameters decreased significantly (p-values between 0 and 0.01) at 3 months to +0.33±0.27 D sphere, -0.50±0.24 D cylinder and 0.10±0.05 microns HOA and were stable at the next follow-ups. Induced spherical equivalent was around zero at all visits. The changes in HOA were mainly due to trefoil aberration.

**Conclusions:**

Linear corneal incisions do not change the spherical power but can induce significant values of astigmatism and trefoil aberration in the cornea. However, these changes revert fully or partially to preoperative values by the third month after surgery and remain stable with time.

## Introduction

In ophthalmologic surgical procedures, mainly with implantation of intraocular lenses, (IOLs), such as cataract surgery and refractive surgery with phakic IOLs, corneal incisions flatten the perpendicular corneal meridian inducing surgically induced astigmatism (SIA) that depends on size[1], location[2,3] and design[4] of the incisions. Currently, the use of injector systems[5] eases the insertion of foldable IOLs through incisions of 3 mm or below, although some rigid phakic IOLs[6] still needs longer incisions of around 6 mm. Previous studies have compared the values of SIA with different sizes of incisions[1–3,7–14] and some of them[1–3,9,10,14] have also evaluated the long-term evolution. In follow-ups between 1 week and 1 month after cataract surgery the mean values of SIA are around 0.50, 0.75 and 1.0 for 2, 3 and 5-mm sizes of incisions respectively. However, there is a high variability between studies and patients, 95% confidence intervals can achieve values of ±1 D. Data at 3 and 6 months show a reduction of initial values of SIA but it is not still clear at what moment the change lets to be significant. In former studies, the SIA has been usually measured as the only refraction parameter that change with corneal incision, although changes in sphere power could be also induced by the incisions. Small increments of positive power in sphere have been reported[12] at 1-month follow-up, but no previous works have measured the long-term changes of both refraction components: sphere and astigmatism. On the other hand, corneal incisions also increase the values of the root mean square (RMS) of higher order aberrations (HOA), some previous studies[15–18] have reported a significant change in trefoil aberration that increases as the corneal incision is larger[19,20]. In these studies, post-surgery data were only collected once during the first six months, so there is currently not information about the long-term evolution of HOA.

In this context, the purpose of this study is to measure and evaluate for one year the values of refraction (sphere, astigmatism and spherical equivalent) and HOA induced by a clear corneal incision of 3.2 mm in eyes with cataract surgery.

## Materials and methods

All clinical examinations and surgery were performed at the Ophthalmology Department, Virgen de la Arrixaca Hospital, Murcia, Spain. The hospital's ethics committee approved this study, which followed the tenets of the Declaration of Helsinki. After receiving an explanation of the nature and possible consequences of the surgery, all patients provided informed consent which also included the authorization to have data from their medical records used in research.

### Patients and surgical technique

Twenty-seven eyes, 15 rights and 12 lefts, of different patients with age ranging from 51 to 79 (mean of 66.7±7.7) years, underwent cataract surgery. All surgeries were performed by the same surgeon (J.M.M.). A linear clear incision was created at temporal side in all eyes using a slit knife with a blade width of 3.2 mm and the paracentesis of 1 mm was located 100- to 120-degrees away from it. Next, 0.5 mL of a dispersive ophthalmic viscosurgical device (OVD) (sodium hyaluronate 3.0%–chondroitin sulfate 4.0% [Viscoat]) was placed in the anterior chamber. A capsulorhexis of 5.5 mm was created and the cataractous lens was extracted with the stop-and-chop phacoemulsification technique, after which 10 mg/mL of a cohesive OVD (sodium hyaluronate 1.0% [Healon]) was placed in the anterior chamber. Then, a 3-piece IOL was implanted in the capsular bag and the incision was not sutured. The residual OVD was aspirated using a bimanual technique. The corneal incision was hydrated at the end of the surgery. A steroidal antiinflammatory and antibiotic were applied to the eye.

### Follow-up visits and optical estimations

Corneal topography was measured 1 week before surgery and 2 weeks (w), 1, 2, 3 and 6 months (m), and 1 year (y) after surgery. From Placido-based corneal topographer (Atlas, software version 1.0.1.0, Carl Zeiss Meditec AG) measurements, the wavefront aberration (WA) of the anterior cornea, expressed as Zernike coefficients, was estimated by ray tracing through the corneal surface (Zemax Development Corp.) realigning the corneal wavefront aberration to the pupil center[21,22]. To monitor differences in relative values of spherical power, the focal length of the cornea was calculated as the distance that minimizes the root-mean-square spot size at the image plane.

The calculated corneal WA was based on the average of at least 4 different topographies in every case. Low-order Zernike coefficients Z3, Z4 and Z5 for 3-mm pupil diameter were used to estimate the corneal refraction. The refraction changes induced by the incisions were calculated as the difference between corneal refraction before and after (in each follow-up) the incision. The corneal refraction was defined as the spherical power and its astigmatism. To estimate accurately the subtractions of refractions, we combined the cylinders using a vector analysis with the formulas of obliquely crossed cylinders given by:

$$tg2b=\frac{c_{2}sen2a}{c_{1}+c_{2}\;\cos\;2a}\;c=\frac{c_{2}sen2a}{sen2b}\;s=\frac{c_{1}+c_{2}-c}{2}$$ (1)

where c_1_ and c_2_ are the cylinders combined, a is the minimum angle between axes, b and c are the axis orientation and the value of outcome cylinder, and s is the outcome sphere that has to be added to the sphere values of the sphero-cylindrical forms combined. For each follow-up, the induced refraction, cylinder and residual sphere, was calculated as the subtraction of follow-up cylinder (c_1_) minus pre-surgery cylinder, and the sphere as the subtraction of follow-up and pre-surgery values plus the residual sphere from cylinders combinations. The corneal refractions were expressed as sphero-cylindrical formula with negative cylinder. Negative values of induced cylinder indicated an increase of pre-surgery value and positive values a reduction.

The changes of HOA induced by the corneal incisions were estimated as the difference between post and pre-surgery Zernike coefficients calculated up to 5th order for 4-mm pupil diameter. The induced RMS of all HOA (HOA RMS), trefoil coefficients (Trefoil RMS) and coma coefficients (Coma RMS) were also estimated.

The posterior surface of the cornea was not considered because previous studies reported too low values of astigmatism and HOA, both in eyes with and without corneal incision, with respect to the anterior surface[23,24].

The statistical analysis was performed using the software R Core Team (R Foundation for Statistical Computing, Vienna, Austria, 2016). From individual measurements, the mean values of optical parameters were calculated with their intersubject errors expressed as standard deviation, errors bars in the graphs. Differences between follow-ups were obtained by means of the Student’s t-test for two paired samples with 2-tailed distribution, and p-values <0.05 were considered statistically significant. Furthermore, linear regression and Pearson correlation coefficient (r) were used to assess the correlation between kerametric parameters and optical changes induced by the incisions.

## Results

Two weeks after surgery, the values of astigmatism induced by the corneal incisions ranged between 0.25 and 1.50 (mean -0.77±0.32) D, but in 22 of the 27 eyes the changes were below 1.00 D. Although the induced sphere ranged from -0.02 to +1.12 D, the combination with the cylinder provided low values of induced spherical equivalent in every eye, between -0.32 and +0.56 D.

**Fig 1:**
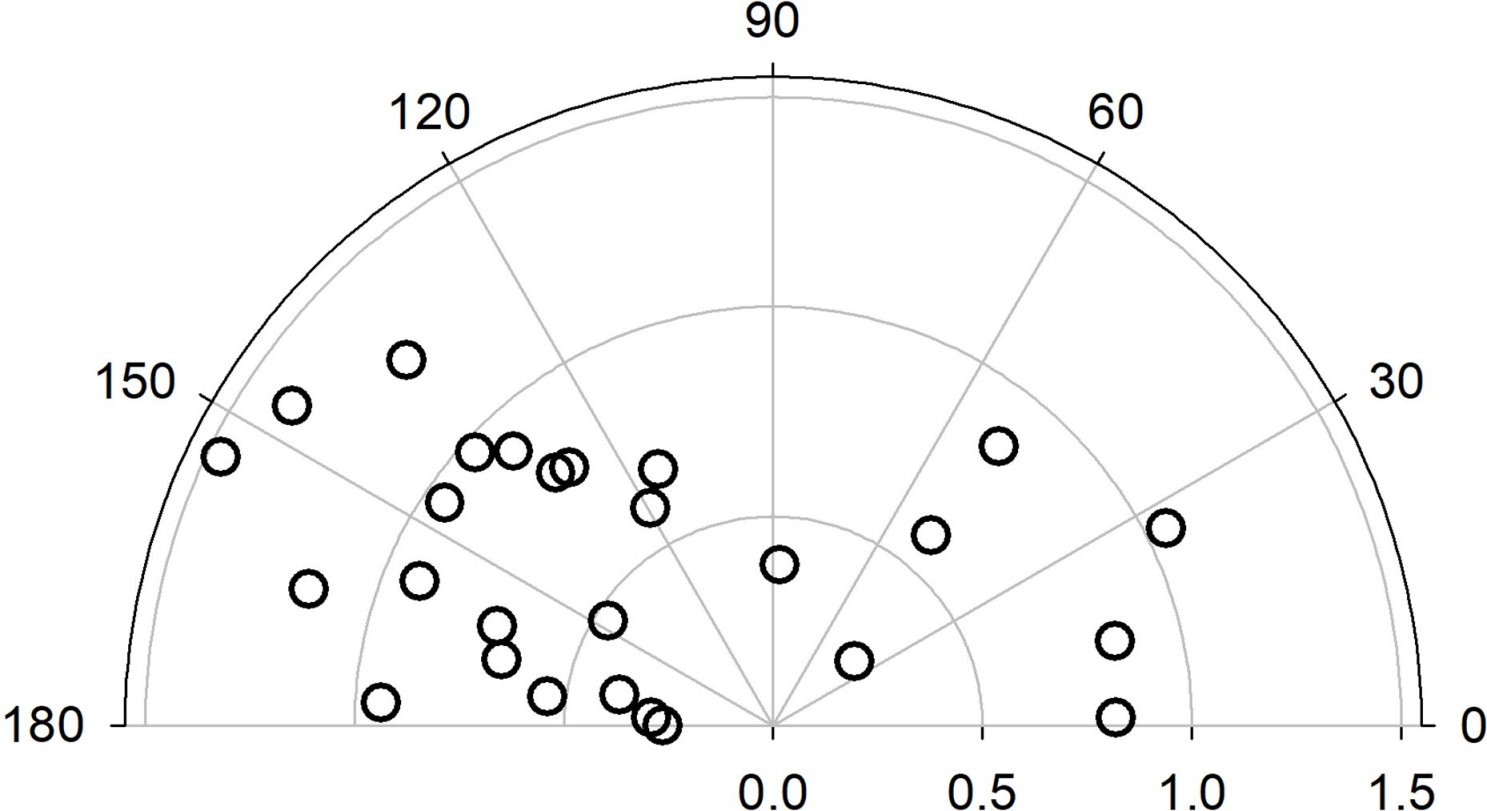
Polar values of 2-weeks post-surgery induced cylinder in cornea.

As expected, the temporal incisions induced astigmatism with the axis of the negative cylinder orientated around the horizontal meridian 0-180º. In practically all eyes, except three, the axis ranged within the intervals [120-180º] and [0-60º], but there was a high variability in the orientation independently on values of astigmatism (Fig 1).

**Fig 2:**
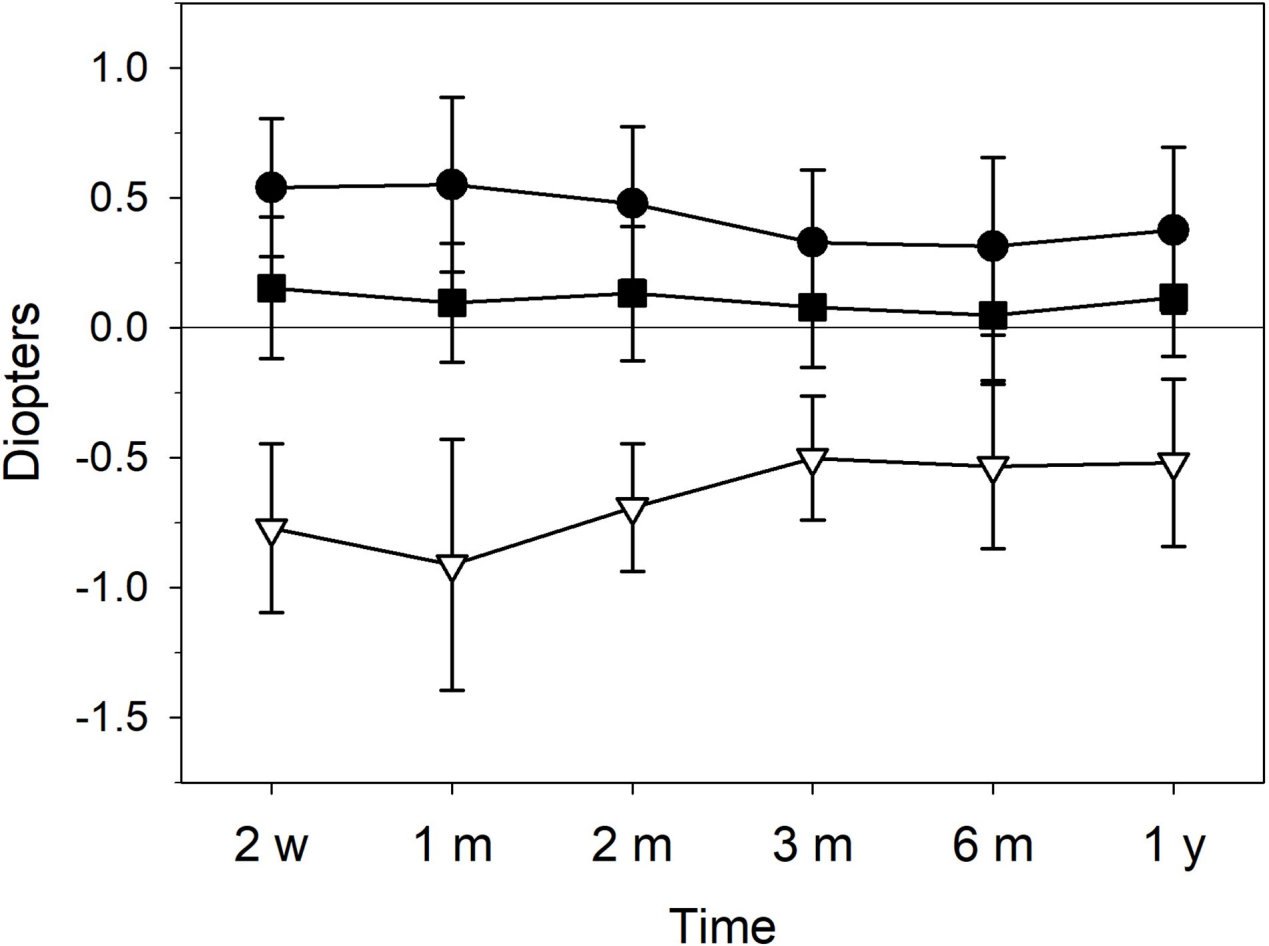
Mean values of induced refraction components of the cornea: sphere (circles), cylinder (triangles) and spherical equivalent (squares), at all follow-ups.

**Fig 3:**
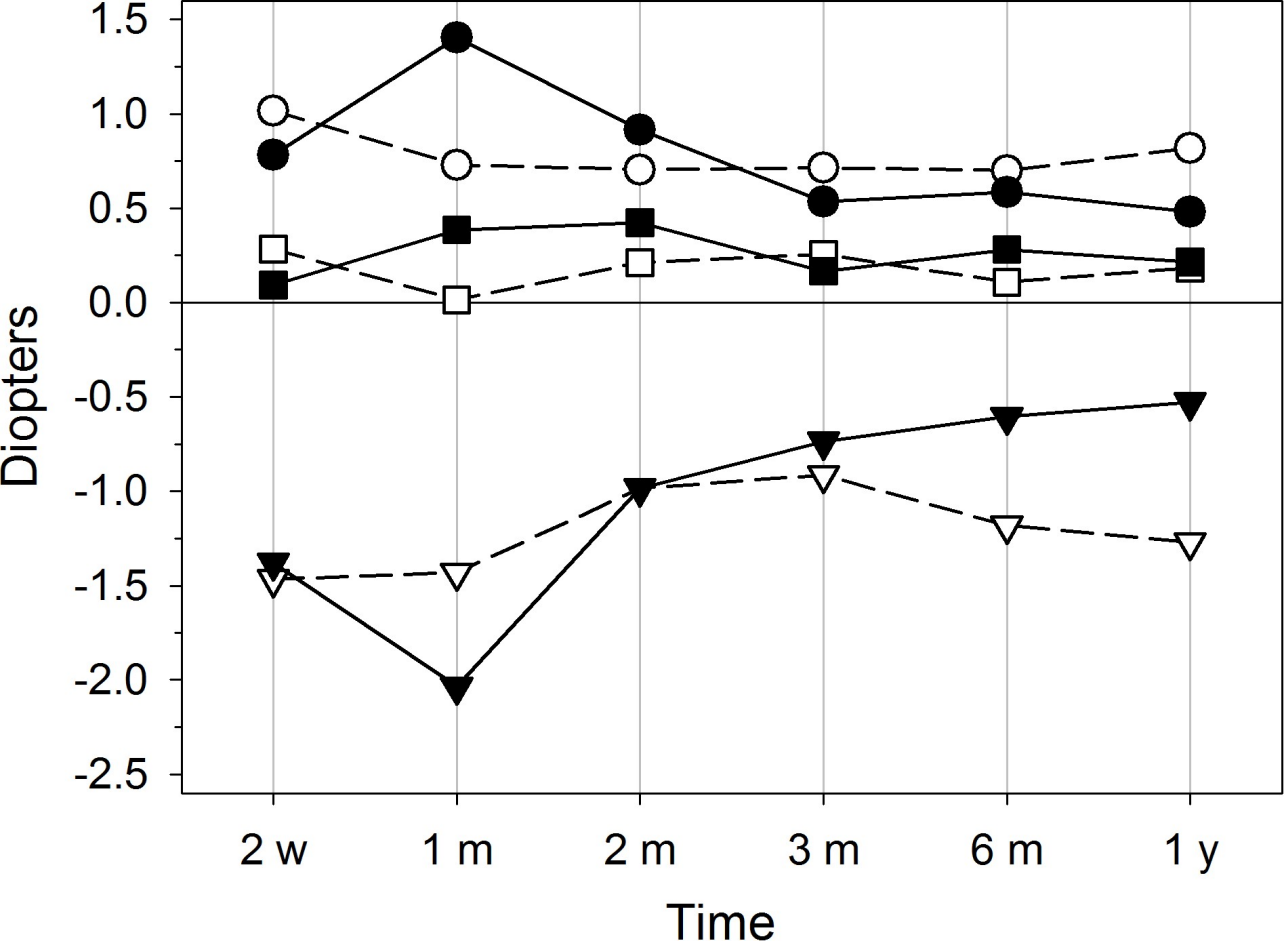
Temporal evolution of induced refraction components of the cornea: sphere (circles), cylinder (triangles) and spherical equivalent (squares), in two right eyes of different patients: S1 (black symbols) and S2 (white symbols).

As shown in Fig 2, the mean values of induced sphere, cylinder and spherical equivalent at 2 weeks after surgery were +0.54±0.27, -0.77±0.32 and +0.15±0.27 D respectively. There were no significant changes at 1- and 2-months follow-ups, except induced cylinder that decreased significantly between the first and second months (p-value < 0.05) from -0.91±0.48 D to -0.69±0.25 D. From the second to the third month, the mean values of all components of the refraction fell significantly (p-value < 0.05) to +0.33±0.27 D sphere, -0.50±0.24 D cylinder and +0.08±0.23 D spherical equivalent and remained stable at the next follow-ups. However, the evolution of induced refraction in time depends on each patient. As an example, Fig 3 displays the evolution of two eyes (S1, S2) with similar initial values of induced sphere, cylinder and spherical equivalent, around +0.90, -1.40 and +0.10 D respectively. The three components of the refraction in S1 increased at 1-month follow-up and then decreased progressively, mainly the cylinder up to -0.50 D, while in S2 the parameters suffered less changes, indeed the cylinder only decreases up to -1.20 D one year after surgery.

**Fig 4:**
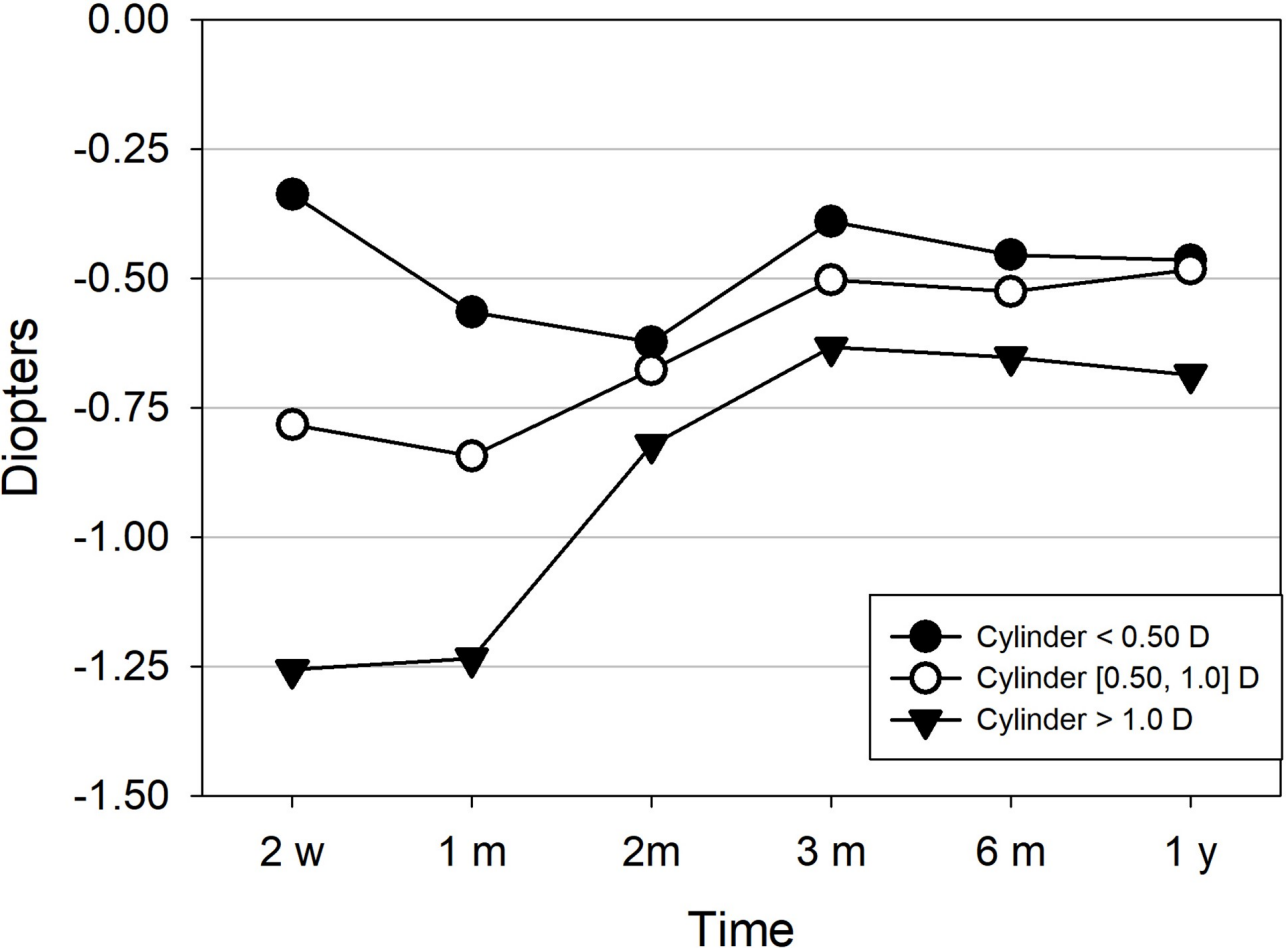
Mean values of induced cylinder for eyes grouped according 2-weeks post-surgery induced cylinder in cornea: less than 0.5 D (black circles. Sample: 6 eyes), between 0.5 and 1.0 D (white circles. Sample: 16 eyes) and higher than 1.0 D (triangles. Sample: 5 eyes), at all follow-ups.

The relative reduction in time of induced cylinder depended on initial values. Fig 4 shows the mean values of eyes grouped in three groups with 2-weeks induced cylinder: (1) less than 0.50 D, (2) between 0.50 and 1.00 D and (3) higher than 1.00 D. There were no relevant changes (p-value > 0.1) for the year in the group 1. In the other two groups, the induced cylinder decreased significantly from the 1st to the 3rd month (p-value < 0.05). In group 2, the mean reduction was about a third of initial values, from -0.78±0.14 D at 2-weeks to -0.50±0.25 D at 3-months follow-up, while in group 3, the reduction was about a half, from -1.26±0.17 D to -0.63±0.21 D. In the next follow-ups, 6 months and 1 year, the values remained stable.

**Fig 5:**
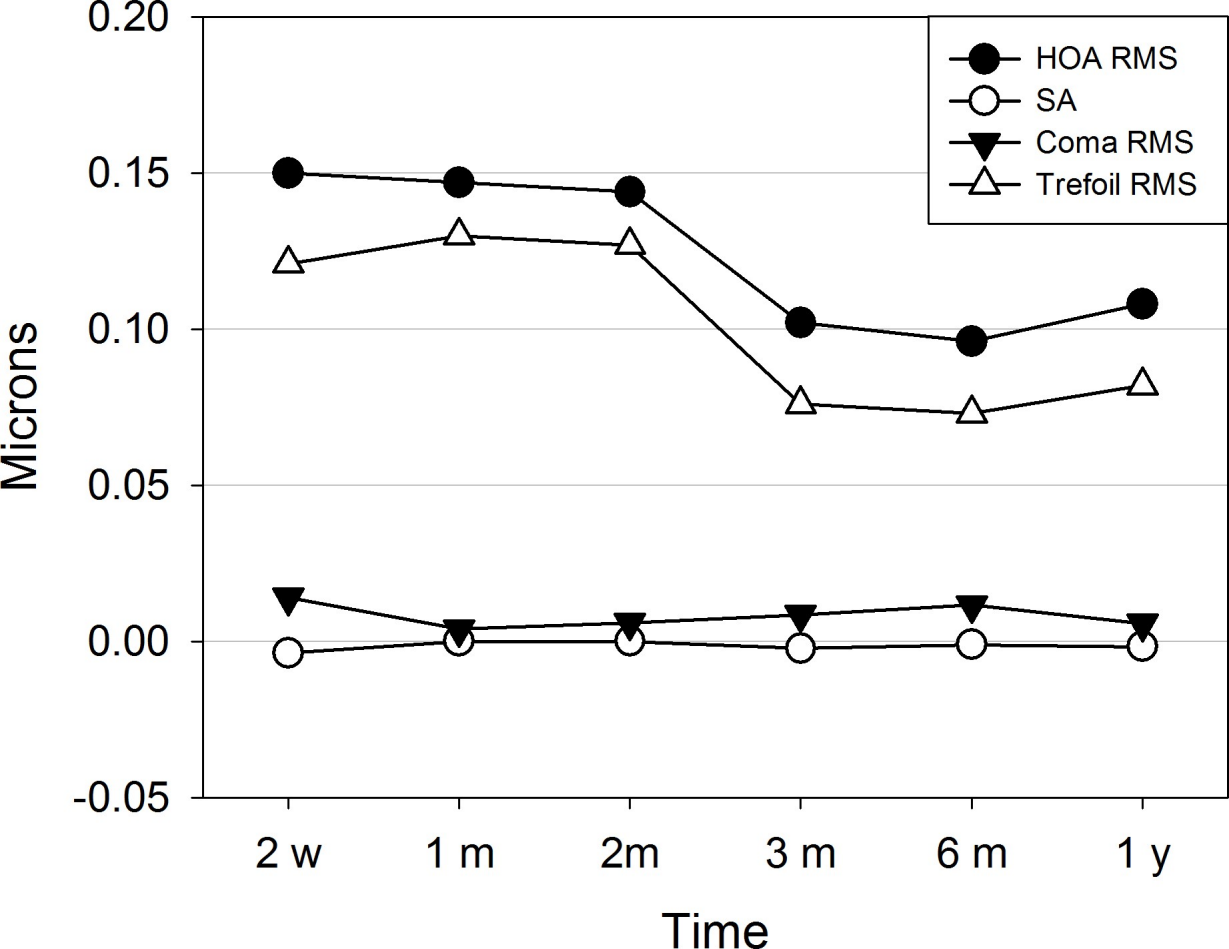
Mean values of induced root-mean-square of higher order aberrations (HOA RMS), spherical aberration (SA), root-mean-square of coma Zernike coefficients (Coma RMS) and root-mean-square of trefoil Zernike coefficients (Trefoil RMS). Maximum values of standard deviations (SD) are: 0.08, 0.02, 0.05 and 0.08 microns for HOA RMS, SA, Coma RMS and Trefoil RMS respectively. 4-mm pupil diameter.

Regarding to the evolution of corneal HOA (Fig 5), the incision did not induce significant variations in Zernike coefficients (Z), except in trefoil aberration. The mean induced values of trefoil coefficients, Z6 and Z9, were -0.10±0.09 and -0.06±0.09 microns at 2-weeks follow-up and dropped to -0.07±0.07 and -0.02±0.07 microns respectively at 3-months follow-up, although the reduction was only significant in Z9 (p-value < 0.05). Therefore, Trefoil RMS decreased significantly between 2 and 3-months follow-ups, from 0.13±0.07 to 0.07±0.05 microns. The combination of trefoil aberration with small values of coma, RMS around 0.01 microns in all follow-ups, and negligible contribution of other aberrations, such as spherical aberration (SA), yielded the same temporal behaviour of total aberrations expressed as the HOA RMS whose values fell significantly from 0.15±0.08 at 2-weeks to 0.10±0.05 microns at 3-months follow-up.

**Fig 6:**
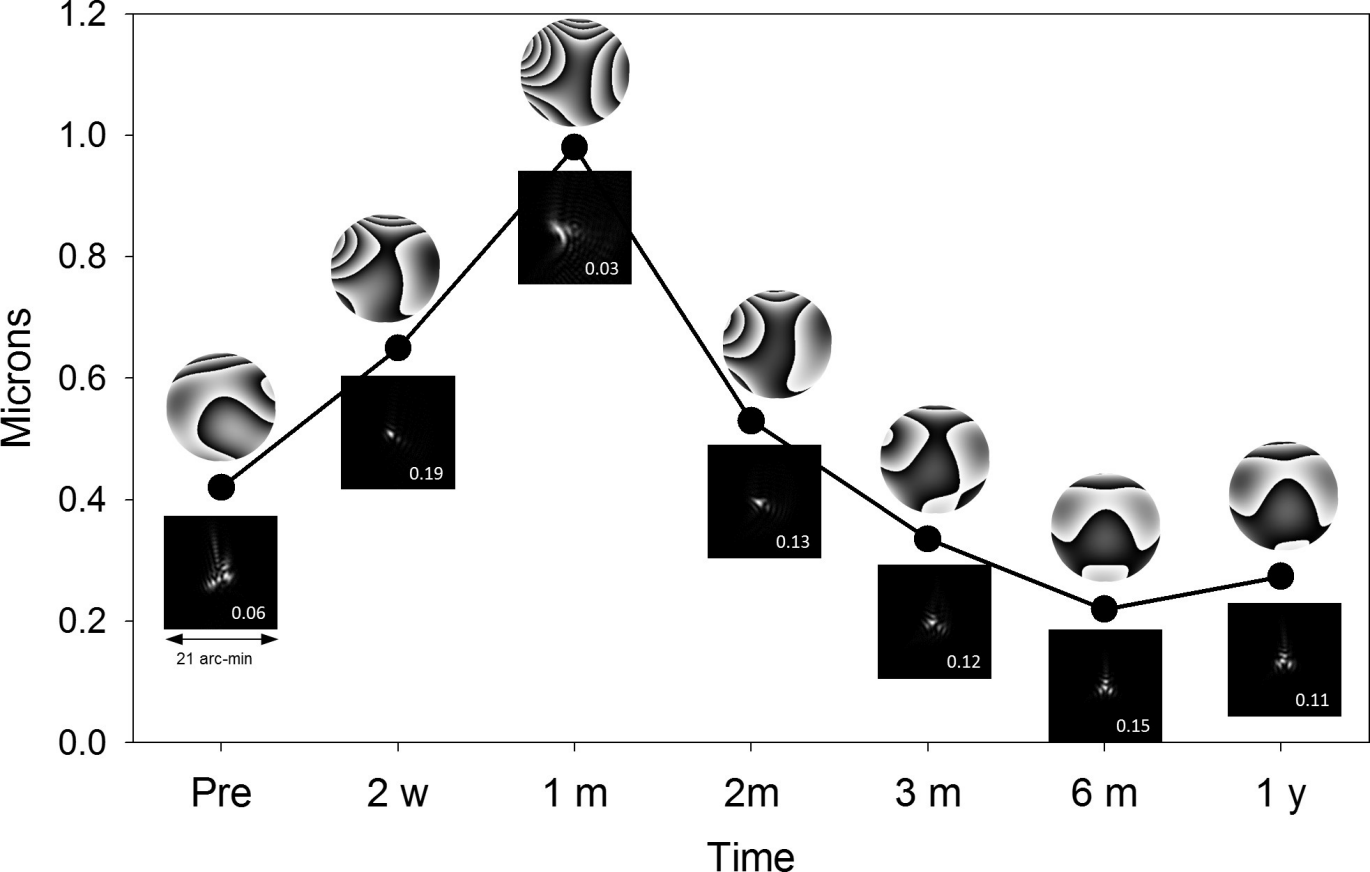
RMS as function of time and corresponding modulus 2π representations of the WA and the associated PSFs with the Strehl ratio values considering astigmatism and HOA (defocus Zernike coefficient 4 set to zero) in the right eye of patient S1. 4-mm pupil diameter.

The combination of induced astigmatism and HOA, mainly trefoil, determined the optical quality of the eyes and its evolution in time. As an example, Fig 6 shows the RMS of Zernike coefficients corresponding to astigmatism and HOA in the right eye of patient S1 at pre-surgery and post-surgery follow-ups. The defocus Zernike coefficient was set to zero because the absolute value of spherical power of corneal refraction cannot be estimated in the standard refraction formulation. In any way, the changes in the spherical equivalent were too small (Fig 3) to affect significantly the optical quality. In addition to RMS values, Fig 6 also displays the WA maps, the associated point-spread functions (PSF) and the values of Strehl ratio (SR) calculated as the quotient between the intensity peak in the system’s PSF and the diffraction-limited PSF. In this case, the RMS values increased with surgery from 0.42 to 0.65 microns, mainly due to an increment in the Zernike coefficients of astigmatism, Z3 and Z5, and trefoil, Z6. However, the change of sign of Z3 in combination with an increment of positive values of Z6 improved the image quality expressed as higher values of SR. But the continuous increase of these aberrations from 2 weeks to 1 month caused a deterioration of the image quality that improved in the next follow-ups, 2 and 3 months, due to a significant reduction of astigmatism and trefoil. For the rest of the year, the WA remained stable with an image quality even better than before surgery due to lower values of coefficient Z3.

## Discussion

In cataract surgery the size of incision is getting smaller even below 2 mm, but longer incisions are still performed in the implantation of some type of pseudophakic IOLs[25] and specially in refractive surgery with phakic IOLs[6,26], where the incision lengths are usually 3 or 6 mm for foldable and non-foldable lenses respectively. It is well-known that corneal incisions change the corneal shape inducing new values of astigmatism and other optical aberrations mainly trefoil. Some surgeons use corneal incisions to relax the steepest meridian and correct corneal astigmatism[27,28]. So, the incision increases or decreases initial values of corneal astigmatism depending on its orientation with respect to the steepest meridian. In both scenarios, it is necessary to know the evolution in time to manage the refraction and possible refractive corrections.

Our measurements and estimations of changes in refraction and aberrations induced by 3.2-mm corneal incision for one-year follow-up allow to detect when the cornea achieves the optical stability. The values of induced astigmatism vary from negligible values to 1.50 D. No significant changes in time are observed in eyes with induced astigmatism below 0.5 D. In average, if induced astigmatism is higher than 0.5 D, corneas change during the first 3 months following surgery. The changes happen in both components of refraction, sphere and cylinder, and the combination of both remain the values of spherical equivalent around zero at all visits. The stabilization of corneal shape at 3 months is also confirmed by the trefoil aberration, the only HOA induced by the incision, that is significantly reduced only between the second and the third month.

Our results agree and complete previous studies where mean values of astigmatism induced by incisions of 2.8–3.5 mm ranged from 0.6 to 1.1 D[1,3,7–9] in agreement with our mean value of 0.8 D. In both previous and current studies, cylinder drops to values around 0.5 D at 3 or 6-months follow-ups[1,3,9,13]. Current study increases the number and the time of follow-ups for one year allowing to conclude that in most patients the stable refraction is achieved at 3 months after 3.2-mm corneal incision, but the spherical equivalent is always close to zero.

On the other hand, previous studies have reported a neural adaptation to own subject’s aberrations[29,30] that could explain the high values of visual quality in subjects with significant amounts of HOA[31] and astigmatism[32] up to 0.5 D. In our study, the values of astigmatism and trefoil induced by the surgery and the changes for the first 3 months yield continuous changes in the corneal WA during this period in many patients. So, the neural adaptation is disrupted by the surgery and a new adaptation process could not be possible until the third month when the corneal WA starts to be stable.

As conclusion, linear corneal incisions of 3.2-mm do not change the spherical power but induce values of astigmatism up to 1.5 D and trefoil aberration up to 0.3 microns (4-mm pupil diameter). However, these changes revert fully or partially to preoperative values by the third month after surgery and remain stable with time.

## Supporting information

S1 Table Individual values of Zernike coefficients (3-mm pupil size) up to 4^th^ order at all follow-ups. Fifth order coefficients were negligible.
(XLSX)
